# The independent role of fine particulate matter and genetic liability on cognition in older adults

**DOI:** 10.1186/s12991-025-00559-9

**Published:** 2025-04-03

**Authors:** Shu-Fen Liao, Ta-Chien Chan, Mei-Hsin Su, Mei-Chen Lin, Chi-Shin Wu, Chun-Chieh Fan, Shi-Heng Wang

**Affiliations:** 1 Department of Medical Research, Wan Fang Hospital, Taipei Medical University, Taipei, Taiwan; 2 School of Public Health, College of Public Health, Taipei Medical University, Taipei, Taiwan; 3 Research Center for Humanities and Social Sciences, Academia Sinica, Taipei, Taiwan; 4 Institute of Public Health, School of Medicine, National Yang Ming Chiao Tung University, Taipei, Taiwan; 5 Department of Public Health, College of Public Health, China Medical University, Taichung, Taiwan; 6 Department of Psychiatry, Virginia Institute for Psychiatric Behavioral Genetics, Virginia Commonwealth University, Richmond, VA USA; 7 National Center for Geriatrics and Welfare Research, National Health Research Institutes, 35, Keyan Road, Zhunan Town, Miaoli County 350, Miaoli, Taiwan; 8 Department of Psychiatry, National Taiwan University Hospital, Yunlin branch, Douliu, Taiwan; 9 Center for Population Neuroscience and Genetics, Laureate Institute for Brain Research, Tulsa, OK USA; 10 Department of Radiology, School of Medicine, University of California San Diego, La Jolla, CA USA; 11 Department of Medical Research, China Medical University Hospital, China Medical University, Taichung, Taiwan

**Keywords:** Air pollution, Alzheimer’s disease, Major depression, PM_2.5_, Polygenic risk score, Schizophrenia, Taiwan biobank

## Abstract

**Background:**

Genetic susceptibility to mental health and cognitive traits, as well as air pollution, significantly impact cognition. The interplay between polygenic liability and fine particulate matter (PM_2.5_) remains unclear due to the limited number of large-scale studies in Asia. This study utilized the Taiwan Biobank, a nationwide community-based database, to investigate the main and modified effect of PM_2.5_ on individuals’ polygenic susceptibility in cognition.

**Methods:**

Polygenic risk score (PRS) for cognitive performance (CP PRS), Alzheimer’s disease (AD PRS), schizophrenia (SCZ PRS), and major depression (MDD PRS) were computed representing genetic susceptibility for an individual. *APOE* genotype was classified into E3/E3, E3/E4, and E4/E4. The five-year average concentration of PM_2.5_ from satellite images was used for defining environmental exposure. Cognitive performance was evaluated via the Mini-Mental State Examination (MMSE) score. The association between personal genetic susceptibility, PM_2.5_, and cognitive performance was examined using multilevel linear regression with the adjustment of age, sex, batch effect, and population stratification effect. The gene-environment synergism was examined with the inclusion of product term of PM_2.5_ and PRS in the multivariate model.

**Results:**

Our analyses included 25,593 participants from 164 townships. Participants exposed to higher PM_2.5_ concentrations had a lower MMSE score (Beta=-0.0830 corresponding to a 1 µg/m^3^ increase in PM_2.5_ concentration, 95% CI, -0.0973 to -0.0688, p-value < 0.0001). After controlling for PM_2.5_ concentration, CP PRS (Beta = 0.1729, 95% CI, 0.1470 to 0.1988, p-value < 0.0001), SCZ PRS (Beta=-0.0632, 95% CI, -0.0891 to -0.0374, p-value < 0.0001), and AD PRS (Beta=-0.0321, 95% CI, -0.0580 to -0.0062, p-value = 0.0153) were associated with MMSE score. After further examination of gene-environment synergism, no interaction effect was identified, indicating different mechanism of PM_2.5_ and genetic liability to influence cognitive performance.

**Conclusions:**

Human polygenic loading and PM_2.5_ may impact cognition via an independent pathway. A prevention strategy targeting air pollution reduction may effectively improve the cognitive performance. Multiple exposures and their influences on the long-term change of cognition were required in future research.

**Supplementary Information:**

The online version contains supplementary material available at 10.1186/s12991-025-00559-9.

## Background

Population aging is the most pervasive demographic trend worldwide. Aging impairs human cognitive ability, resulting in socioeconomic and health burdens [1, 2]. A decline in cognitive performance often presents before the onset of several psychotic disorders [3, 4]. Therefore, understanding the determinant factors associated with cognition may help generate public health prevention strategies and mitigate the detrimental effects of cognitive ability in older populations.

An increasing number of studies targeting on the influence of air pollution on human mental health, including depression, anxiety and cognitive impairment [5–7]. Exposure to air pollutants was postulated to harm the central nervous system and affect psychological functioning by direct toxicity or inducing neuro-inflammation [5, 8]. Fine particulate matter (PM_2.5_) has been found to have a negative effect on general cognition among adults > 40 years [1]. A meta-analysis presented a significant decrease in general cognition for the increase of PM_2.5_ concentration [1].

General cognitive ability is attributed to genetic factors, with a high heritability of 50–70% [9–11]. Polygenic risk score (PRS), combining the effects of trait-susceptible genetic variants identified in a genome-wide association study (GWAS), was utilized recently to comprehensively evaluate the genetic liability of a phenotype. A higher cognitive polygenic capacity was significantly correlated with better cognition in a population of European ancestry [11, 12]. Genetic correlations between general cognition and psychotic syndromes was also reported [13, 14]. A high PRS for schizophrenia or major depression (MDD) was associated with poor general cognitive ability and spatial visualization [3, 15–18]. The *Apolipoprotein E* (*APOE*) locus was known associated with Alzheimer’s disease (AD) and cognitive decline, with a higher risk for epsilon 4 (E4) allele [11, 12, 19]. In addition, the PRS for AD combining common variants except for *APOE* was associated with reduced hippocampal volume and cognitive impairment in late life [20, 21].

The interplay of individual genetic effects and environmental factors in a complex phenotype was important [16]. However, the literature is still limited. Two small studies conducted in China have examined interactions between MDD PRS and air pollution for cognitive impairment through the alteration of brain function via DNA methylation or the influence of stress-related corticol network function [8, 22, 23]. A child study revealed the antagonistic effect of *APOE* status, AD PRS and air pollution through the change of brain structural morphology [19]. In addition, large-scale genetic studies were mainly conducted in Western countries, resulting in a knowledge gap regarding the role of polygenic susceptibility, air pollutants, and their potential interaction on cognition in Asian populations.

This study aimed to utilize the Taiwan Biobank (TWB), a nationwide community-based database, to specifically identify the genetic liability for cognition in the Taiwan population and evaluate the main and modified effect of exposure to PM_2.5_ on the population. We expect the study findings provide new insights into intervention strategies to maintain human cognition and alleviate subsequent disease burdens.

## Materials and methods

### Study population

The study population included participants in the Taiwan Biobank [24, 25]. The Taiwan Biobank was designed to be a nationwide community-based study, recruiting cancer-free individuals aged 30–70 years. It is the largest government-supported biobank, including more than 120,000 participants. Detailed information on the participants’ socio-demographic characteristics, lifestyle and behavior, personal health status, family history of disease, physical and biological status, and genetic features were included. This study was approved by the Central Regional Research Ethics Committee of China Medical University, Taichung, Taiwan (CRREC-108-30).

### Fine particulate matter (PM_2.5_)

PM_2.5_ was assessed using high-resolution, specified air pollution datasets maintained by the Atmospheric Composition Analysis Group of Washington University in St. Louis. These were global estimates of ground-level ambient PM_2.5_, gridded at approximately 1 km × 1 km [26]. We used zonal statistic function with the township boundary of Taiwan by ArcGIS (ArcMap, version 10.3; ESRI Inc., Redlands, CA, USA) to compute the annual concentration of PM_2.5_ in each township. Participants’ exposure to PM_2.5_ was specified by their residential address and was defined by the five-year average before the year of baseline determination. We further categorized the exposure to PM_2.5_ in quartiles (< 18.7, 18.7‒22.2, 22.2‒29.1, 29.1+) to elucidate the dose-response association between the level of environmental exposure and cognitive performance.

### Genotyping and PRS

Genome-wide genotyping was performed for 131,048 TWB samples using the customized Taiwan Biobank chip and through the Axiom Genome-Wide Array Plate System (Affymetrix, Santa Clara, CA, USA). Details of quality control and SNP imputation have been previously described [27]. Genetic variants with low call rate (< 5%), low minor allele frequency (< 0.001), or significant deviation from the Hardy-Weinberg equilibrium (*p* < 1E-6) were excluded to reduce genotyping error. Genotype imputation was performed based on the reference panel of 504 East Asian (EAS) (Genomes Project Consortium, 2015) and 973 TWB panel, resulting in a total of 12,601,684 variants with imputation info > 0.7 available. Individuals (i) with genotyping missing rate > 2% in all chromosomes, (ii) EAS descent, and (iii) that are outliers in the heterozygosity/homozygosity test were filtered out. For two participants with the identity by descent sharing coefficients PI-HAT > 0.1875, only one of them was kept for further analysis to prevent cryptic relatedness. To account for population stratification bias, we conducted a principal component analysis and used the top 20 principal components (PCs) as covariates for subsequent analyses [4]. Finally, 106,806 unrelated participants were included in the PRS calculation.

PRS for cognitive performance (CP), AD, schizophrenia (SCZ), and MDD were computed. CP PRS was derived from COGENT consortium (*N* = 35,298) [13] and UK Biobank (*N* = 222,543) [28] of European descent using PRS-CS [29]. AD PRS was derived from a discovery sample of 39,918 cases and 358,140 controls of European descent (excluding 23andMe and proxy cases/controls from UK Biobank) [30] using PRS-CS. Genetic variations for *APOE* were excluded for the calculation of AD PRS to examine the non-*APOE* genetic predisposition to AD [19]. The *APOE* genotypes were classified as E3/E3, E3/E4, and E4/E4 according to rs7412 and rs429358. The Psychiatric Genomics Consortium meta-analysis was used as a discovery sample to identify the risk variants for SCZ and MDD. To optimize PRS prediction, we derived SCZ PRS using PRS-CSx [31] to integrate GWAS in 22,778 cases and 35,362 controls of EAS descent [32] and GWAS in 53,386 cases and 77,258 controls of European descent [33]. We derived MDD PRS using PRS-CSx to integrate GWAS in 246,363 cases and 561,190 controls of European descent [34] and GWAS in 13,042 cases and 88,467 controls of EAS descent [35]. All the PRSs were standardized as a z-distribution. The association between each PRS and their corresponding phenotype among TWB population was presented in Supplementary Table 1, showing satisfactory validity of the derived PRS.

### Socioeconomic covariates

Participants’ education level and the community-level socioeconomic covariates were evaluated here. Individual educational level was categorized into five groups: illiteracy, self-study, or elementary school; junior high school; senior high school or vocational school; university or college; and master’s degree or above. Individual socioeconomic characteristics were defined by the community-level socioeconomic statistics of their residential district, including unemployment rate, median household income, coefficient of variation (CV) of household income, and single-parent rate, from the 2015 Monthly Bulletin of Social Welfare Statistics and the 2015 Monthly Bulletin of Interior Statistics. The administrative district is classified as an urban, suburban, or rural area to reflect the degree of urbanization [36].

### Assessment of cognitive performance

Cognitive performance was evaluated using the Mini-Mental State Examination (MMSE). MMSE is a validated cognitive screening tool for dementia [37]. It quantitatively assesses orientation, memory attention, calculation, and language function, with a 0–30 point score system. A higher MMSE score indicates better cognitive performance. In the Taiwan Biobank study, MMSE was conducted among participants ≥ 60 years.

### Statistical analyses

The distribution of continuous variables is presented using means and standard deviations (SDs), and the distribution of categorical variables is presented using numbers and percentages. The association between personal characteristics, PRS, *APOE* genotype, PM_2.5_, community-level socioeconomic covariates, and cognitive performance was examined using multilevel linear regression to obtain unbiased estimates for individual- and group-level variables. The residential districts were incorporated in multilevel model as the random effect to account for the heterogeneous correlation across groups. Age, sex, batch effect, and the top 20 PCs were included in the multilevel linear model to control for population stratification bias and potential confounding. PM_2.5_, PRS or *APOE* genotype, and variables significantly associated with the MMSE score in the univariate analysis were used for multivariate modeling. The individual and the product term of PM_2.5_ and PRS or *APOE* genotype were included in the multivariate model to examine the gene-environment synergism. The simple slope analysis was further applied for the visualization of PM_2.5_–PRS and PM_2.5_–*APOE* genotype interaction. The stratified analyses for the association between PRS or *APOE* genotype and MMSE score using PM_2.5_ quartiles were conducted to examine whether the effect of PRS or *APOE* genotype on cognitive performance changed with the PM_2.5_ exposure level.

We restricted our analyses to participants whose residential areas were in the administrative districts with sample sizes > 20 to obtain unbiased and precise estimates in slope variances [38, 39]. The significance level was set at 0.05. All statistical analyses were performed using the SAS statistical software package (version 9.4 for Windows; SAS Institute Inc., Cary, NC, USA).

## Results

### Study participants

Among the TWB participants, 26,674 individuals aged > 60 years took examination of cognition via MMSE. After excluding 1,081 participants residing in 157 townships (each had a sample size of ≤ 20), a total of 25,593 participants from 164 townships were included in the analyses. The mean MMSE score was 27.52 (Table 1). Table 1 presents the distribution of participants’ baseline characteristics. In the older adult population, the mean age was 63.01 years, 61.18% were female, and most (75.42%) had higher educational levels than senior (vocational) high school. The mean PM_2.5_ concentration was 23.48 µg/m^3^, with the quartile cut-off points 18.7, 22.2, and 29.1 µg/m^3^. The mean community-level socioeconomic covariates were 3.78% for the unemployment rate, 641.16 thousand New Taiwan Dollars (NTD) for median household income, 211.61 for CV household income, and 7.35% for the single-parent rate. More than 68% of participants resided in the urban area. Most (81.66%) of TWB participants carried homozygous *APOE* E3 alleles and only a small portion (0.77%) of them carried homozygous *APOE* E4 alleles.

**Table 1:** The distribution of baseline characteristics and the univariate associations with MMSE score

	*N* = 25,593	Univariate analyses	
	Mean (SD)/ n(%)	Beta (95% CI)	p-value
Age, years	63.01 (3.48)	-0.0702 (-0.0784, -0.0620)	**< 0.0001**
Sex			
Male	9934 (38.82)	Ref.	
Female	15,659 (61.18)	-0.2450 (-0.3033, -0.1867)	**< 0.0001**
Education ^a^			
Illiteracy, self-study, or elementary school	3382 (13.22)	Ref.	
Junior high school	2908 (11.37)	1.5377 (1.4327, 1.6426)	**< 0.0001**
Senior (vocational) high school	8024 (31.37)	2.4593 (2.3735, 2.5451)	**< 0.0001**
University (college)	9638 (37.68)	3.0101 (2.9252, 3.0951)	**< 0.0001**
Master and above	1629 (6.37)	3.2328 (3.1060, 3.3596)	**< 0.0001**
CP PRS ^b^	0.0 (1.0)	0.2268 (0.1985, 0.2551)	**< 0.0001**
SCZ PRS ^b^	0.0 (1.0)	-0.0520 (-0.0805, -0.0236)	**0.0003**
MDD PRS ^b^	0.0 (1.0)	0.0260 (-0.0025, 0.0544)	0.0735
AD PRS ^b^	0.0 (1.0)	-0.0475 (-0.0760, -0.0190)	**0.0011**
*APOE* genotype ^c^			
E3/E3	17,917 (81.66)	Ref.	
E3/E4	3854 (17.57)	-0.0182 (-0.0990, 0.0627)	0.6598
E4/E4	170 (0.77)	-0.2145 (-0.5657, 0.1366)	0.2311
PM_2.5_ (µg/m^3^)	23.48 (5.51)	-0.0830 (-0.0973, -0.0688)	**< 0.0001**
PM_2.5_ level			
< 18.7	6398 (25.00)	Ref.	
18.7 to 22.2	6432 (25.13)	-0.1132 (-0.2263, 0.000025)	0.0501
22.2 to 29.1	6389 (24.96)	-0.1764 (-0.3334, -0.01933)	**0.0277**
29.1+	6374 (24.91)	-0.7839 (-0.9611, -0.6066)	**< 0.0001**
Unemployment rate (%)	3.78 (0.08)	-0.4242 (-2.0373, 1.1890)	0.6063
Median household income (1,000 NTD)	641.16 (81.95)	0.0051 (0.0040, 0.0063)	**< 0.0001**
CV of household income	211.61 (263.08)	0.0006 (0.0002, 0.0010)	**0.0040**
Single-parent rate (%)	7.35 (2.05)	0.0291 (-0.0235, 0.0817)	0.2782
Urbanization			
1 (Urban)	17,602 (68.78)	Ref.	
2 (Sub-urban)	7451 (29.11)	-0.7342 (-0.9346, -0.5337)	**< 0.0001**
3 (Rural)	540 (2.11)	-0.7450 (-1.1316, -0.3584)	**0.0002**
MMSE score	27.52 (2.38)	‒	‒

Abbreviations: MMSE, Mini-Mental State Examination; PRS, polygenic risk score; SD, standard deviation; AD, Alzheimer’s disease; CP, cognition performance; SCZ, schizophrenia; MDD, major depression disorder; PM_2.5_, fine particulate matter; CV, coefficient of variation; *APOE*, apolipoprotein E

^a^ available in 25,581 participants for the total population

^b^ standardized to z-distribution with mean of zero and standard deviation of 1.0

^c^ available in 21,941 participants for the total population

### PM_2.5_, genetic liability, and cognitive performance

The univariate analyses of baseline characteristics and MMSE score are presented in Table 1. Older age (Beta=-0.0702, 95% CI, -0.0784 to -0.0620, p-value < 0.0001) and female sex (Beta=-0.2450, 95% CI, -0.3033 to -0.1867, p-value < 0.0001) were significantly associated with decreased MMSE scores. Individuals with educational levels of senior (vocational) high school or higher had better cognitive performance than those who were illiterate, self-studying, or educated in elementary school. For community-level socioeconomic variables, the higher the median income (Beta = 0.0051, 95% CI, 0.0040 to 0.0063, p-value < 0.0001) or CV household income (Beta = 0.0006, 95% CI, 0.0002 to 0.0010, p-value = 0.0040), the better the cognitive performance. The unemployment and single-parent rates are not significantly associated with MMSE score. A significantly lower cognitive performance was presented among individuals living in sub-urban (Beta=-0.7342, 95% CI, -0.9346 to -0.5377, p-value < 0.0001) and rural area( Beta=-0.7450, 95% CI, -1.1316 to -0.3584, p-value = 0.0002).

PM_2.5_ concentration was significantly associated with poor cognitive performance (Table 1). Participants exposed to higher PM_2.5_ concentrations had a lower MMSE score (Beta=-0.0830 corresponding to a 1 µg/m^3^ increase in PM_2.5_ concentration, 95% CI, -0.0973 to -0.0688, p-value < 0.0001). PM_2.5_ concentrations between 22.2 and 29.1 µg/m^3^ (Beta=-0.1764, 95% CI, -0.3334 to -0.0193, p-value = 0.0277) and higher than 29.1 µg/m^3^ (Beta=-0.7839, 95% CI, -0.9611 to -0.6066, p-value < 0.0001) were associated with lower MMSE scores compared to PM_2.5_ concentration < 18.7 µg/m^3^. The dose-response relationship had statistical significance (p-value for trend test < 0.0001).

Genetic liability was crucial in participants’ cognitive performance (Table 1). CP PRS (Beta = 0.2268, 95% CI, 0.1985 to 0.2551, p-value < 0.0001) was positively correlated with the MMSE score, while AD PRS (Beta=-0.0475, 95% CI, -0.0760 to -0.0190, p-value = 0.0011) and SCZ PRS (Beta=-0.0520, 95% CI, -0.0805 to -0.0236, p-value = 0.0003) were negatively correlated with the MMSE score. MDD PRS (Beta = 0.0260, 95% CI, -0.0025 to 0.0544, p-value = 0.0735) showed a non-significant association with cognitive performance. *APOE* genotype also showed no significant relevance to cognition, with Beta=-0.0182 (95% CI, -0.0990 to 0.0627, p-value = 0.6598) for E3/E4 genotype and Beta=-0.2145 (95% CI, -0.5657 to 0.1366, p-value = 0.2311) for E4/E4 genotype compared to E3/E3 genotype.

We performed multivariable analyses to examine the association between PM_2.5_, PRS, and MMSE scores (Table 2). After adjusting for population stratification (top 20 PCs), batch effect, and confounding factors, CP PRS and PM_2.5_ were significantly associated with cognitive performance. A 0.0285 (adjusted Beta=-0.0285, 95% CI, -0.0392 to -0.0178, p-value < 0.0001) decrease in MMSE score corresponded with a 1 µg/m^3^ increase in PM_2.5_ concentration, and a 0.1729 (adjusted Beta = 0.1729, 95% CI, 0.1470 to 0.1988, p-value < 0.0001) increase in MMSE score corresponded with a one SD increase in CP PRS. SCZ PRS and PM_2.5_ exhibited a negative association with cognitive performance, presenting a lower MMSE score for higher PM_2.5_ concentration (adjusted Beta=-0.0281, 95% CI, -0.0389 to -0.0174, p-value < 0.0001) and polygenic loading (adjusted Beta=-0.0632, 95% CI, -0.0891 to -0.0374, p-value < 0.0001). The negative effect of AD PRS (adjusted Beta=-0.0321, 95% CI, -0.0580 to -0.0062, p-value = 0.0153) was significant for MMSE score as PM_2.5_ was included in the multivariate model. The effect of MDD PRS remained non-significant when the multiple-adjusted model was applied. The multivariate results of *APOE* genotype on cognition was presented in Table 3, showing non-significant effect for MMSE (E3/E4 vs. E3/E3: adjusted Beta=-0.0174, 95% CI, -0.0906 to 0.0558, p-value = 0.6421; E4/E4 vs. E3/E3: adjusted Beta=-0.2827, 95% CI, -0.6004 to 0.0350, p-value = 0.0811).

**Table 2:** Multivariate associations of PM_2.5_, PRS and MMSE score

	CP PRS		SCZ PRS		MDD PRS		AD PRS	
	Adjusted Beta (95% CI) ^a^	p-value	Adjusted Beta (95% CI) ^a^	p-value	Adjusted Beta (95% CI) ^a^	p-value	Adjusted Beta (95% CI) ^a^	p-value
**PM**_**2.5**_	-0.0285 (-0.0392, -0.0178)	**< 0.0001**	-0.0281 (-0.0389, -0.0174)	**< 0.0001**	-0.0280 (-0.0387, -0.0172)	**< 0.0001**	-0.0276 (-0.0388, -0.0168)	**< 0.0001**
**PRS**	0.1729 (0.1470, 0.1988)	**< 0.0001**	-0.0632 (-0.0891, -0.0374)	**< 0.0001**	0.0152 (-0.0107, 0.0410)	0.2503	-0.0321 (-0.0580, -0.0062)	**0.0153**
Age	-0.0454 (-0.0531, -0.0378)	**< 0.0001**	-0.0450 (-0.0527, -0.0373)	**< 0.0001**	-0.0450 (-0.0527, -0.0373)	**< 0.0001**	-0.0450 (-0.0527, -0.0373)	**< 0.0001**
Sex								
Male	Ref.		Ref.		Ref.		Ref.	
Female	0.1321 (0.0766, 0.1876)	**< 0.0001**	0.1369 (0.0813, 0.1926)	**< 0.0001**	0.1358 (0.0802, 0.1915)	**< 0.0001**	0.1319 (0.0761, 0.1878)	**< 0.0001**
Education								
Illiteracy, self-study, or elementary school	Ref.		Ref.		Ref.		Ref.	
Junior high school	1.4837 (1.3793, 1.5882)	**< 0.0001**	1.4940 (1.3893, 1.5987)	**< 0.0001**	1.4935 (1.3887, 1.5983)	**< 0.0001**	1.4902 (1.3852, 1.5952)	**< 0.0001**
Senior (Vocational) high school	2.3692 (2.2824, 2.4560)	**< 0.0001**	2.3859 (2.2989, 2.4730)	**< 0.0001**	2.3835 (2.2964, 2.4706)	**< 0.0001**	2.3816 (2.2943, 2.4689)	**< 0.0001**
University (College)	2.9229 (2.8346, 3.0112)	**< 0.0001**	2.9660 (2.8777, 3.0544)	**< 0.0001**	2.9613 (2.8728, 3.0497)	**< 0.0001**	2.9592 (2.8705, 3.0478)	**< 0.0001**
Master and above	3.1418 (3.0118, 3.2717)	**< 0.0001**	3.2006 (3.0705, 3.3307)	**< 0.0001**	3.1959 (3.0658, 3.3261)	**< 0.0001**	3.1896 (3.0591, 3.3201)	**< 0.0001**
Median household income	0.0014 (0.0004, 0.0025)	**0.0081**	0.0015 (0.0004, 0.0025)	**0.0085**	0.0014 (0.0004, 0.0025)	**0.0088**	0.0014 (0.0003, 0.0025)	**0.0097**
CV household income	0.0001 (-0.0001, 0.0004)	0.2716	0.0001 (-0.0001, 0.0004)	0.2639	0.0002 (-0.0001, 0.0004)	0.2516	0.0002 (-0.0001, 0.0004)	0.2270
Urbanization								
1 (Urban)	Ref.		Ref.		Ref.		Ref.	
2 (Sub-urban)	-0.1725 (-0.3393, -0.0056)	**0.0428**	-0.1733 (-0.3415, -0.0051)	**0.0434**	-0.1721 (-0.3402, -0.0041)	**0.0447**	-0.1774 (-0.3458, -0.0089)	**0.0390**
3 (Rural)	-0.1577 (-0.4642, 0.1487)	0.3131	-0.1636 (-0.4722, 0.1450)	0.2987	-0.1637 (-0.4721, 0.1447)	0.2981	-0.1676 (-0.4767, 0.1415)	0.2879

Abbreviations: MMSE, Mini-Mental State Examination; PRS, polygenic risk score; SD, standard deviation; AD, Alzheimer’s disease; CP, cognition performance; SCZ, schizophrenia; MDD, major depression disorder; CV, coefficient of variation; Beta, regression coefficient.schizophrenia; MDD, major depression disorder; CV, coefficient of variation; Beta, regression coefficient

^a^ Batch and 20 principal components were also included in multivariate models for adjustment

**Table 3:** Multivariate associations of PM_2.5_, *APOE* genotype and MMSE score

	Adjusted Beta (95% CI) ^a^	*p*-value
**PM**_**2.5**_	-0.0235 (-0.0345, -0.0124)	**< 0.0001**
***APOE*** **genotype**		
E3/E3	Ref.	
E3/E4	-0.0174 (-0.0906, 0.0558)	0.6421
E4/E4	-0.2827 (-0.6004, 0.0350)	0.0811
Age	-0.0453 (-0.0537, -0.0370)	**< 0.0001**
Sex		
Male	Ref.	
Female	0.1275 (0.0674, 0.1877)	**< 0.0001**
Education		
Illiteracy, self-study, or elementary school	Ref.	
Junior high school	1.4761 (1.3633, 1.5889)	**< 0.0001**
Senior (Vocational) high school	2.3851 (2.291 L, 2.4790)	**< 0.0001**
University (College)	2.9575 (2.8622, 3.0528)	**< 0.0001**
Master and above	3.2197 (3.0793, 3.3600)	**< 0.0001**
Median household income	0.0014 (0.0003, 0.0025)	**0.0098**
CV household income	0.0002 (-0.0001, 0.0004)	0.2369
Urbanization		
1 (Urban)	Ref.	
2 (Sub-urban)	-0.1803 (-0.3489, -0.0117)	**0.0361**
3 (Rural)	-0.2138 (-0.5268, 0.0992)	0.1806

Abbreviations: MMSE, Mini-Mental State Examination; CV, coefficient of variation; Beta, regression coefficient; *APOE*, apolipoprotein E

^a^ Batch and 20 principal components were also included in multivariate models for adjustment

### Independent effect of genetic liability and PM_2.5_

**Fig. 1:**
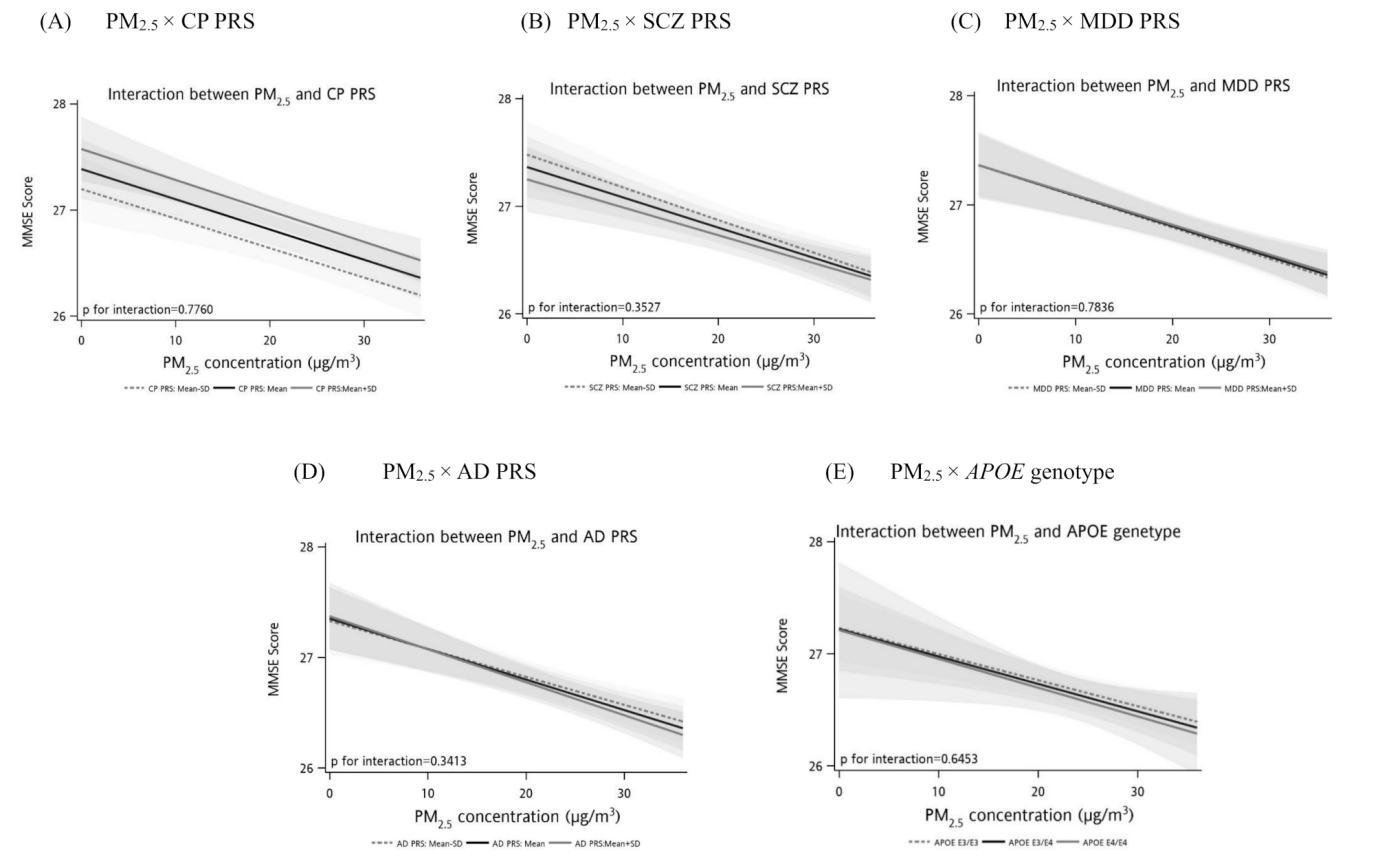
Interaction between PM_2.5_ and PRS or *APOE* genotype on MMSE score. (**A**) PM_2.5_**×** CP PRS. (**B**) PM_2.5_**×** SCZ PRS. (**C**) PM_2.5_**×** MDD PRS. (**D**) PM_2.5_**×** AD PRS. (**E**) PM_2.5_**×***APOE* genotype

The interaction between PM_2.5_ and PRS or *APOE* genotype was presented in Figure and Supplementary Tables 2, 3. The p-value for the product term of PM_2.5_-CP PRS (Fig. 1A), PM_2.5_-SCZ PRS (Fig. 1B), PM_2.5_-MDD PRS (Fig. 1C), PM_2.5_-AD PRS (Fig. 1D), and PM_2.5_-APOE genotype (Fig. 1E) was 0.7760, 0.3527, 0.7836, 0.3413, and 0.8452, respectively. It indicated the independent effect of PM_2.5_ and genetic liability associated with cognitive performance. Similar results were obtained when the categorized PM_2.5_ level was instead included in the multivariate model (Supplementary Tables 2–3) and stratified analyses (Supplementary Tables 4, 5).

## Discussion

This large-scale, population-based study was conducted to unravel the role of genetic liability, air pollution, and their interplay on cognition. Increased PM_2.5_ concentration, lower CP PRS, higher SCZ PRS and AD PRS were associated factors to cognitive impairment. No gene-environment interactions were identified in the study, indicating the distinct mechanisms of polygenic loading or PM_2.5_ in cognition causation.

The large-scale meta-analysis, including 86 studies from Europe, North America, Asia and Australia, indicated a PM_2.5_-cognition association of -0.02 (95% CI: -0.03, -0.00, p-value < 0.05) in middle-to-older populations [1]. PM_2.5_ was related to poor cognitive function in Chinese population [2]. In the present study, the hazard of PM_2.5_ on cognition was replicated in the Taiwanese population, with a 0.0285 (adjusted Beta=-0.0285, 95% CI, -0.0392 to -0.0178, p-value < 0.0001) decrease in MMSE score for the increase of PM_2.5_ concentration.

This study identified the independent effect of CP and SCZ liability on cognitive performance and it agrees with several Western studies [3, 11, 12]. The bidirectional relationship between schizophrenia and cognition was also identified in a genome-wide association meta-analysis [17]. Several studies have suggested a genetic correlation between cognition and AD through the involvement of *APOE* or genetic liability to AD [13, 19–21, 40, 41]. However, we observed a significant effect of AD PRS but not *APOE* genotype among Taiwnese population. This may be due to the limited sample size of *APOE* homozygous E4 genotype in our study. Evidence for the association between MDD PRS and cognition remains controversial. The genome-wide interaction study revealed a significant but distinct effect of MDD liability on different sub-domains of cognition, including reaction time, immediate memory, and global cognition [15], but a null effect was presented in the study.

Our study did not detect an interaction between polygenic effect and PM_2.5_, implying that a distinct mechanism of genetic and environmental factors may be involved. It was postulated that PM_2.5_ affects cognition via the degeneration of neurons or neuro-inflammation. Neurons in the olfactory bulb (OB) and hippocampus were considered critical for cognitive ability, including learning, memory, and sensory performance [42]. Calderón-Garcidueñas et al. reported ultrafine particles in human OB periglomerular neurons via the inhalation of air pollutants [43], causing direct contact with exogenous chemicals, which impacts neurogenesis and develops AD [44]. Meanwhile, PM_2.5_ induces immune response for the activation of macrophages and inflammatory cytokines, leading to the accumulation of reactive oxygen species (ROS) and inflammation. Inflammatory compounds and ROS in circulation may spread to blood-brain barrier [2], leading to the induction of neuro-inflammation and the impairment of cognitive function. In-vitro studies have reported poor microglial cell survival for high-dose PM_2.5_ due to neuro-inflammation and ROS production [45, 46]. Apart from the modulation of neurogenesis from PM_2.5_, the potential pathway of genetic architecture was through the physical change of brain structure, such as gray matter atrophy [12]. The cognition-associated genes dominate innate diversity between people and influence individual susceptibility, responding to alterations in brain structure over time, eventually impacting human cognition [12].

Our study had several strengths. First, this is the first community-based study to elucidate the complex roles of polygenic characteristics and PM_2.5_ on general cognition rather than clinical features, such as dementia. It may be more helpful to generate public health practice early during cognitive dysfunction and postpone the development of psychological disorders. Second, considering the diversity of genetic profiles and environmental factors worldwide, our study was advantageous for demonstrating their importance in Asian population and complementing the knowledge gap between countries. Third, we applied PRS to comprehensively summarize the subtle effect of genetic variants on cognition. The trans-ancestry PRS was computed for SCZ PRS and MDD PRS to integrate different genetic contributions across ethnic populations [31] to enhance the accuracy of effect size estimation.

However, our study had some limitations. First, it was a cross-sectional observation. Each participant’s residence was only investigated at baseline, resulting in possible misclassification and underestimation of PM_2.5_ exposure. Second, only PM_2.5_ was considered in the study. A mixed exposure model with the inclusion of exercise and other pollutants such as nitrogen oxide [1], sulfur [47], road traffic noise [48], or second-hand smoking [2] was suggested to be more representative of real-life situations. Third, we included participants aged more than 60 for analyses because MMSE was only measured in a subsample of Taiwan Biobank. The generalizability was limited and further studies targeting on the association of PM_2.5_ and genetic liability with cognition among younger aged population may be required. Fourth, the MMSE score was not in a normal distribution (Kolmogorov-Smirnov p-value < 0.01) and the normality assumption was violated for regression modeling. However, normality examination has been reported invalid with large sample sizes [49]. Therefore, according to the Central Limit Theorem [50, 51] and the simulation study [52], even without adherence to normality assumption, parameter estimates would be in normal distribution and unbiased estimates would be still obtained in our large-scale study. Fifth, the common variants-based PRS did not capture the total genetic liability. Using cross-ancestry GWAS results to derive PRS may lead to a low prediction [53] because genetic architecture may differ across populations. Most large-scale GWAS have been performed in individuals of European ancestry, with only a few reported in individuals of East Asian ancestry, e.g., SCZ. Although a moderate-scale GWAS for MDD has been performed in East Asian ancestry, the limited sample size restricted the improvement of PRS prediction in East Asian populations [54] and subsequently limited the statistical power for cross-trait PRS association test. Further large-scale genetic research in individuals of diverse ancestries is needed.

## Conclusion

In conclusion, independent associations were revealed between human polygenic loading, PM_2.5_, and cognitive performance. A prevention strategy targeting air pollution reduction may effectively improve cognition. Effects of multiple exposures and their influnces on the long-term change of cognition were await discovery in future research.

## Electronic supplementary material

Below is the link to the electronic supplementary material.

Supplementary Material 1

## Data Availability

No datasets were generated or analysed during the current study.

## Abbreviations

AD: Alzheimer’s disease
APOE: Apolipoprotein E
CV: Coefficient of variation
CP: Cognition performance
EAS: East Asian
GWAS: Genome-wide association study
MDD: Major depressive disorder
MMSE: Mini-Mental State Examination
NTD: New Taiwan Dollars
OB: Olfactory bulb
PM2.5: Fine particulate matter
PRS: Polygenic risk score
PCs: Principal components
ROS: Reactive oxygen species
SCZ: Schizophrenia
SDs: Standard deviations
TWB: Taiwan Biobank
