# Supplementary material for: The independent role of fine particulate matter and genetic liability on cognition in older adults
Source: Ann Gen Psychiatry. 2025 Apr 3;24:20. doi: 10.1186/s12991-025-00559-9 (PMC11969746; doi:10.1186/s12991-025-00559-9)
Supplement: Supplementary file 1 — Supplementary Material 1 [file 12991_2025_559_MOESM1_ESM.docx]

**Supplementary information**

**The independent role of fine particulate matter and genetic liability on cognition in older adults**

Supplementary Table 1. Associations between PRS and the corresponding phenotype in the Taiwan Biobank

|  | Adjusted OR (95% CI) ^a^ | p-value |
| --- | --- | --- |
| SCZ PRS vs. Schizophrenia | 1.64 (1.52, 1.77) | **<0.0001** |
| MDD PRS vs. Major depression | 1.18 (1.16, 1.20) | **<0.0001** |
| AD PRS vs Alzheimer's disease | 1.21 (1.10, 1.32) | **<0.0001** |

^a^ adjusted for age, sex, batch, and 20 principal components

Supplementary Table 2. Independent and interactive effects of PM_2.5_ and PRS on MMSE score

|  | CP PRS | | SCZ PRS | | MDD PRS | | AD PRS | |
| --- | --- | --- | --- | --- | --- | --- | --- | --- |
|  | Adjusted Beta (95% CI) ^a^ | p-value | Adjusted Beta (95% CI) ^a^ | p-value | Adjusted Beta (95% CI) ^a^ | p-value | Adjusted Beta (95% CI) ^a^ | p-value |
| **PM_2.5_** | -0.0285 (-0.0392, -0.0178) | **<0.0001** | -0.0281 (-0.0389, -0.0174) | **<0.0001** | -0.0279 (-0.0387, -0.0172) | **<0.0001** | -0.0276 (-0.0383, -0.0168) | **<0.0001** |
| **PRS** | 0.1888 (0.0764, 0.3012) | **0.0010** | -0.1152 (-0.2277, -0.0027) | **0.0448** | -0.0004 (-0.1140, 0.1133) | 0.9952 | 0.0215 (-0.0918, 0.1347) | 0.7105 |
| **PM_2.5_ × PRS** | -0.0007 (-0.0053, 0.0040) | 0.7760 | 0.0022 (-0.0025, 0.0069) | 0.3527 | 0.0007 (-0.0041, 0.0054) | 0.7836 | -0.0023 (-0.0070, 0.0024) | 0.3413 |
| **PM_2.5_ level** |  |  |  |  |  |  |  |  |
| <18.7 | Ref. |  | Ref. |  | Ref. |  | Ref. |  |
| 18.7 to 22.2 | 0.0879 (-0.0119, 0.1878) | 0.0842 | 0.0841 (-0.0161, 0.1843) | 0.1000 | 0.0842 (-0.0160, 0.1844) | 0.0996 | 0.0878 (-0.0123, 0.1883) | 0.0868 |
| 22.2 to 29.1 | 0.0740 (-0.0528, 0.2008) | 0.2527 | 0.0746 (-0.0529, 0.2022) | 0.2516 | 0.0741 (-0.0534, 0.2016) | 0.2544 | 0.0770 (-0.0508, 0.2048) | 0.2376 |
| 29.1+ | -0.2886 (-0.4324, -0.1448) | **<0.0001** | -0.2872 (-0.4318, -0.1425) | **0.0001** | -0.2839 (-0.4286, -0.1393) | **0.0001** | -0.2824 (-0.4274, -0.1374) | **0.0001** |
| **PRS** | 0.1516 (0.1005, 0.2028) | **<0.0001** | -0.0913 (-0.1423, -0.0403) | **0.0005** | 0.0136 (-0.0381, 0.0652) | 0.6069 | -0.0336 (-0.0853, 0.0181) | 0.2021 |
| **PM_2.5_ level × PRS** |  | 0.6843 |  | 0.6030 |  | 0.2017 |  | 0.6523 |
| PM_2.5_ (<18.7) **×** PRS | Ref. |  | Ref. |  | Ref. |  | Ref. |  |
| PM_2.5_ (18.7 to 22.2) **×** PRS | 0.0444 (-0.0280, 0.1168) | 0.2292 | 0.0259 (-0.0462, 0.0979) | 0.4816 | 0.0119 (-0.0610, 0.0848) | 0.7490 | 0.0092 (-0.0634, 0.0817) | 0.8047 |
| PM_2.5_ (22.2 to 29.1) **×** PRS | 0.0244 (-0.0484, 0.0972) | 0.5110 | 0.0435 (-0.0291, 0.1161) | 0.2406 | -0.0405 (-0.1131, 0.0321) | 0.2746 | 0.0223 (-0.0510, 0.0957) | 0.5512 |
| PM_2.5_ (29.1+) **×** PRS | 0.0168 (-0.0555, 0.0891) | 0.6485 | 0.0437 (-0.0294, 0.1167) | 0.2413 | 0.0377 (-0.0355, 0.1109) | 0.3132 | -0.0240 (-0.0971, 0.0492) | 0.5209 |

Abbreviations: MMSE, Mini-Mental State Examination; PRS, polygenic risk score; SD, standard deviation; AD, Alzheimer's disease; CP, cognition performance; SCZ, schizophrenia; MDD, major depression disorder; PM_2.5_, fine particulate matter; CV, coefficient of variation; Beta, regression coefficient.

^a^ adjusted for age, sex, batch, 20 principal components, education, urbanization, median household income, and CV household income.

Supplementary Table 3. Independent and interactive effects of PM_2.5_ and *APOE* genotype on MMSE score

|  | Adjusted Beta (95% CI) ^a^ | p-value |
| --- | --- | --- |
| **PM_2.5_** | -0.0235 (-0.0347, -0.0122) | **<0.0001** |
| ***APOE* genotype** |  |  |
| E3/E3 | Ref. |  |
| E3/E4 | -0.0510 (-0.3708, 0.2688) | 0.7546 |
| E4/E4 | 0.3437 (-1.0538, 1.7412) | 0.6298 |
| **PM_2.5_ ×*APOE*** |  | 0.6453 |
| PM_2.5_ × *APOE* E3/E3 | Ref. |  |
| PM_2.5_ × *APOE* E3/E4 | 0.0014 (-0.0118, 0.0147) | 0.8322 |
| PM_2.5_ × *APOE* E4/E4 | -0.0263 (-0.0834, 0.0308) | 0.3670 |
| **PM_2.5_ level** |  |  |
| <18.7 | Ref. |  |
| 18.7 to 22.2 | 0.0691 (-0.0450, 0.1832) | 0.2351 |
| 22.2 to 29.1 | 0.0686 (-0.0709, 0.2081) | 0.3352 |
| 29.1+ | -0.2821 (-0.4388, -0.1254) | **0.0004** |
| ***APOE* genotype** |  |  |
| E3/E3 | Ref. |  |
| E3/E4 | -0.0535 (-0.1997, 0.0928) | 0.4735 |
| E4/E4 | -0.0325 (-0.6862, 0.6213) | 0.9224 |
| **PM_2.5_ level × PRS** |  | 0.5783 |
| PM_2.5_ (<18.7) **×** *APOE* E3/E3 | Ref. |  |
| PM_2.5_ (18.7 to 22.2) **×** *APOE* E3/E3 | - |  |
| PM_2.5_ (22.2 to 29.1) **×***APOE* E3/E3 | - |  |
| PM_2.5_ (29.1+) **×** *APOE* E3/E3 | - |  |
| PM_2.5_ (<18.7) **×** *APOE* E3/E4 | Ref. |  |
| PM_2.5_ (18.7 to 22.2) **×** *APOE* E3/E4 | 0.0294 (-0.1752, 0.2339) | 0.7786 |
| PM_2.5_ (22.2 to 29.1) **×** *APOE* E3/E4 | 0.1261 (-0.0830, 0.3352) | 0.2372 |
| PM_2.5_ (29.1+) **×** *APOE* E3/E4 | -0.0006 (-0.2074, 0.2043) | 0.9958 |
| PM_2.5_ (<18.7) **×** *APOE* E4/E4 | Ref. |  |
| PM_2.5_ (18.7 to 22.2) **×** *APOE* E4/E4 | 0.0541 (-0.8765, 0.9846) | 0.9093 |
| PM_2.5_ (22.2 to 29.1) **×** *APOE* E4/E4 | -0.5781 (-1.4720, 0.3157) | 0.2049 |
| PM_2.5_ (29.1+) **×** *APOE* E4/E4 | -0.4072 (-1.3073, 0..4929) | 0.3752 |

Abbreviations: MMSE, Mini-Mental State Examination; PM_2.5_, fine particulate matter; Beta, regression coefficient; CV, coefficient of variation; *APOE*, [apolipoprotein E](https://www.sciencedirect.com/topics/pharmacology-toxicology-and-pharmaceutical-science/apolipoprotein-e).

^a^ adjusted for age, sex, batch, 20 principal components, education, urbanization, median household income, and CV household income.

Supplementary Table 4. Stratified analyses for the association between PRS and MMSE score, by PM_2.5_ level

|  | N (%) | CP PRS | | SCZ PRS | | MDD PRS | | AD PRS | |
| --- | --- | --- | --- | --- | --- | --- | --- | --- | --- |
|  |  | Adjusted Beta  (95% CI) ^a^ | p-value | Adjusted Beta  (95% CI) ^a^ | p-value | Adjusted Beta  (95% CI) ^a^ | p-value | Adjusted Beta  (95% CI) ^a^ | p-value |
| PM_2.5_ level |  |  |  |  |  |  |  |  |  |
| <18.7 | 6398 (25.00) | 0.1530  (0.1049, 0.2011) | **<0.0001** | -0.0927  (-0.1404, -0.0451) | **0.0001** | 0.0128  (-0.0355, 0.0612) | 0.6026 | -0.0302  (-0.0786, 0.0182) | 0.2214 |
| 18.7 to 22.2 | 6432 (25.13) | 0.1890  (0.1353, 0.2426) | **<0.0001** | -0.0670  (-0.1199, -0.0141) | **0.0131** | 0.0302  (-0.0232, 0.0836) | 0.2676 | -0.0190  (-0.0721, 0.0340) | 0.4822 |
| 22.2 to 29.1 | 6389 (24.96) | 0.1736  (0.1225, 0.2247) | **<0.0001** | -0.0438  (-0.0945, 0.0070) | 0.0910 | -0.0273  (-0.0774, 0.0228) | 0.2852 | -0.0147  (-0.0658, 0.0364) | 0.5737 |
| 29.1+ | 6374 (24.91) | 0.1733  (0.1190, 0.2277) | **<0.0001** | -0.0404  (-0.0957, 0.0150) | 0.1526 | 0.0534  (-0.0014, 0.1081) | 0.0561 | -0.0629  (-0.1176, -0.0081) | **0.0244** |

Abbreviations: MMSE, Mini-Mental State Examination; PRS, polygenic risk score; SD, standard deviation; AD, Alzheimer's disease; CP, cognition performance; SCZ, schizophrenia; MDD, major depression disorder; PM_2.5_, fine particulate matter; CV, coefficient of variation; Beta, regression coefficient.

^a^ adjusted for age, sex, batch, 20 principal components, education, urbanization, median household income, and CV household income.

Supplementary Table 5. Stratified analyses for the association between *APOE* genotype and MMSE score, by PM_2.5_ level

| PM_2.5_ level | *APOE* genotype | Adjusted Beta (95% CI) ^a^ | p-value |
| --- | --- | --- | --- |
| <18.7 | E3/E3 | Ref. |  |
|  | E3/E4 | -0.0581 (-0.1953, 0.0791) | 0.4063 |
|  | E4/E4 | -0.0517 (-0.6651, 0.5618) | 0.8689 |
| 18.7 to 22.2 | E3/E3 | Ref. |  |
|  | E3/E4 | -0.0292 (-0.1797, 0.1213) | 0.7035 |
|  | E4/E4 | 0.0010 (-0.6862, 0.7061) | 0.9776 |
| 22.2 to 29.1 | E3/E3 | Ref. |  |
|  | E3/E4 | 0.0729 (-0.0715, 0.2173) | 0.3226 |
|  | E4/E4 | -0.5661 (-1.1544, 0.0223) | 0.0593 |
| 29.1+ | E3/E3 | Ref. |  |
|  | E3/E4 | -0.0543 (-0.2073, 0.0986) | 0.4862 |
|  | E4/E4 | -0.4051 (-1.0517, 0.2415) | 0.2194 |

Abbreviations: MMSE, Mini-Mental State Examination; PM_2.5_, fine particulate matter; CV, coefficient of variation; Beta, regression coefficient; *APOE*, apolipoprotein E.

^a^ adjusted for age, sex, batch, 20 principal components, education, urbanization, median household income, and CV household income
